# Prognostic value of preoperative body mass index for diabetic patients with non-metastasis gastric cancer: a single center experience

**DOI:** 10.1186/s12893-021-01316-x

**Published:** 2021-08-09

**Authors:** Zaisheng Ye, Shenghong Wei, Yi Zeng, Yi Wang, Zhitao Lin, Shu Chen, Yunqing Xie, Qiuhong Zheng, Luchuan Chen

**Affiliations:** 1 Department of Gastrointestinal Surgical Oncology, Fujian Cancer Hospital, and Fujian Medical University Cancer Hospital, No. 420 Fu-ma Road, Jin-An District, Fuzhou, 350014 China; 2 Department of Fujian Provincial Key Laboratory of Tumor Biotherapy, Fujian Cancer Hospital & Fujian Medical University Cancer Hospital, Fuzhou, 350014 Fujian China

**Keywords:** BMI, Type 2 diabetes mellitus, Gastric cancer, X-tile, Survival rate

## Abstract

**Aim:**

This study was designed to investigate the prognostic effect of preoperative body mass index (BMI) for Type 2 diabetes mellitus (T2DM) patients with non-metastasis gastric cancer (GC) who underwent D_2_ gastrectomy.

**Methods:**

T2DM patients with pT_1–4b_N_0–3b_M_0_ GC were retrospectively collected in Department of Gastrointestinal Surgical Oncology, Fujian Cancer Hospital & Fujian Medical University Cancer Hospital from January, 2000 to December, 2010. These patients underwent D_2_ radical resection of the stomach combined with regional lymphadenectomy. Chi-square test was used to analyze unordered categorical variables and ranked data, followed by Kaplan–Meier analysis as well as Cox regression models to detect risk factors for survival outcomes. In addition, the cut-off point was determined by the X-tile program. All analyses were carried out using survival package of R and SPSS Software.

**Results:**

A total of 302 T2DM patients with pT_1–4b_N_0–3b_M_0_ GC were collected and analyzed. The cut-off points of BMI, identified by the X-tile program, was 19 kg/m^2^. Patients with low BMI (< 19 kg/m^2^) had a higher percentage of advanced T stage (T_4a_ and T_4b_), more advanced TNM stage (stage IIIA, IIIB and IIIC), and more elevated level of serum carcinoembryonic antigen (CEA), compared to those with high BMI (> 19 kg/m^2^) (all *P* < 0.05). In the low BMI subgroup, the 5-year overall survival rate was 39.02%, which was as high as 58.11% in the high BMI subgroup (*P* < 0.05). In the multivariate Cox regression model revealed that III_C_ stage (OR = 3.101), N_3b_ stage (OR = 3.113) were the most important prognostic indicators, followed by pretreatment BMI (OR = 2.136).

**Conclusion:**

Low preoperative BMI (< 19 kg/m^2^) was a poor prognostic marker for T2DM patients with pT_1–4b_N_0–3b_M_0_ GC.

## Introduction

Gastric cancer (GC) ranks the second in all cases of cancer-related mortality, accounting for approximately 1 million GC-related deaths per year [1]. D_2_ radical resection of the stomach combined with regional lymphadenectomy has been verified to be the single radical option [2–6]. Although there has been great advance in diagnosis as well as treatment of GC, little progress has been achieved in long-term prognosis. Hence, it is particularly necessary to find a novel prognostic marker, which is noninvasive and accessible before treatment.

Type 2 diabetes mellitus (T_2_DM) has gradually become a growing global public health burden [7]. The prevalence of T_2_DM is up to 8.3% worldwide [8] according to the International Diabetes Federation, which varies in different regions and countries. It is estimated that 552 million people will develop diabetes by 2030 globally [8]. T_2_DM may predispose patients to premature illness and death due to the relevant risks of cardiovascular diseases [9]. The prevalence of T_2_DM has enhanced substantially in recent years, and the presence of T_2_DM has been confirmed to be related with increased risks of multiple malignancies [10]. Moreover, and the relationship between diabetes mellitus (DM) and risks of developing cancers has been examined in numerous meta-analyses.

Obesity is an emerging risk factor for several cancers worldwide, and the relationship between obesity and cancers has been well investigated in various types of malignancies [11–14]. T_2_DM is a multifactorial and chronic group of metabolic disorders characterized by hyperglycemia [7, 15], which is a result of obesity to some extent. In consideration of the relationship between obesity and long-term post-operative outcome in GC patients, several studies have revealed that obesity/overweight may correlate with the long-term outcome.

Body mass index (BMI) is the most commonly used index of body mass [16]. Some authors have suggested a relationship between increased BMI and esophageal and gastric cardia adenocarcinoma [17–19]. Conversely, some studies have demonstrated that high BMI was associated with a good prognosis of GC patients [20, 21]. According to the World Health Organization (WHO) classification system, BMI is generally categorized into the following four grades [22]: underweight (< 18.5 kg/m^2^), normal weight (18.5–24.9 kg/m^2^), overweight (25.0–29.9 kg/m^2^), and obese (≥ 30.0 kg/m^2^). However, it hardly matches the true circumstance for the GC patients with T_2_DM. The role of preoperative BMI on the survival of T_2_DM patients with GC survival remains unclear. Hence, the retrospective study was designed to investigate the effect of preoperative BMI on the survival outcome in T_2_DM patients with non-metastatic GC after D_2_ gastrectomy.

## Methods

### Patients and clinicopathological characteristics

The clinical data of 302 patients with non metastatic diabetes and GC who underwent D_2_ lymph node dissection from January 2000 to December 2012 at the Department of Gastrointestinal Surgery of, Fujian Cancer Hospital & Fujian Medical University Cancer Hospital were retrospectively analyzed similar to the cohort of previous studies [23].

The inclusion criteria were as follows: (1) pathological diagnosis was adenocarcinoma; (2) D_2_ lymphadenectomy (according to the guidelines of the 2010 Japanese Classification of Gastric Cancer and Gastric Cancer Treatment Guidelines edited by the Japanese Gastric Cancer Association [24]; (3) I–III stage (AJCC TNM 7th edition) [25]; (4) preprandial glucose > 7.1 mmo/L.

The exclusion criteria were as follows: (1) > 80 years of age; (2) history of gastrectomy; (3) previous or combined with other cancer; (4) IV stage (AJCC TNM 7th edition; (5) history of neoadjuvant therapy; (6) non R0 resection; (7) mortality within non tumor causes [23].

The patients were conducted followed-up interviews over the telephone. The information regarding the survival status at the last follow-up was collected similar to the cohort of previous studies. The last follow-up was 1 January 2017 [23].

### Statistical analysis

Chi-square test was used for qualitative data. The survival analysis was performed by the Kaplan–Meier and Cox regression methods. The X-tile program was used to determine the optimal cutoff [26]. All analyses were performed with survival package of R (Version 3.2.1) and SPSS (Version 22.0). Prism 5 for Windows (Version 5.01, GraphPad Software) was used to draft the figure of Kaplan–Meier curve. Values of P < 0.05 were considered significant.

## Results

### Identification of BMI cut-off points

**Fig. 1:**
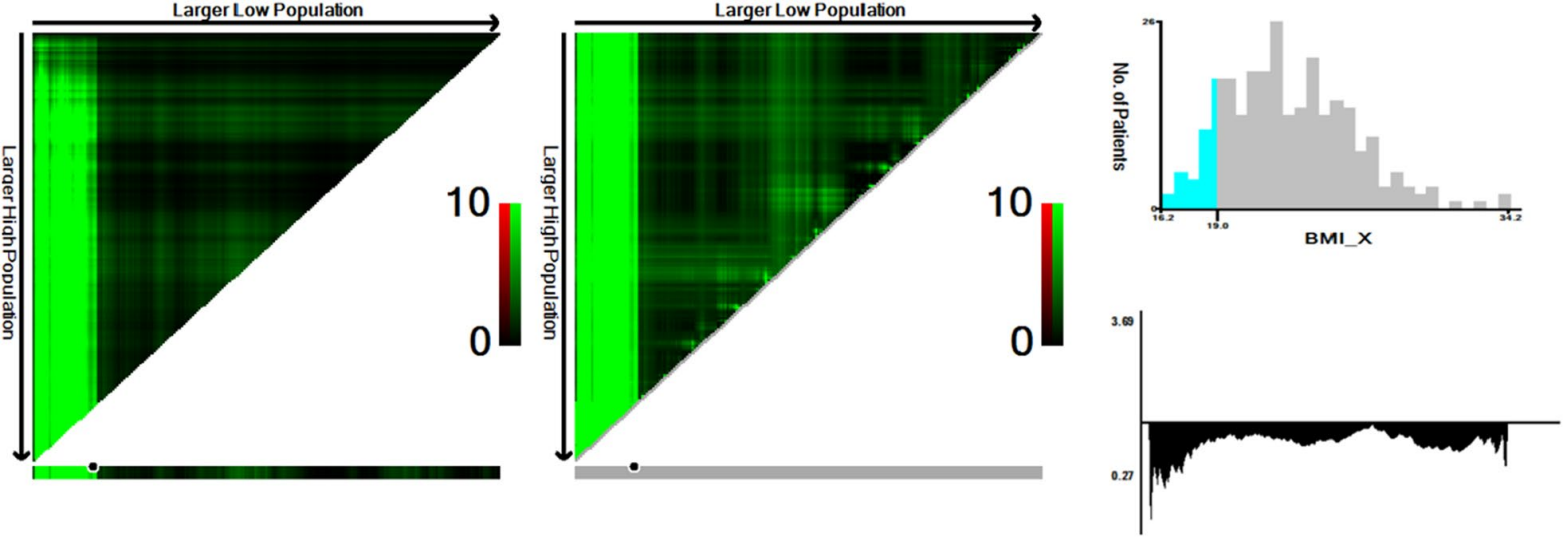
*X-tile* analysis of survival data. X-tile analysis was done on gastric cancer patients with T_2_DM. The optimal cut-point highlighted in the left panels and a Kaplan–Meier plot (right panels). *P* values were determined by using the cut-point defined in the training set and applying it to the validation set. Figures show BMI count divided at the optimal cut-point (19 kg/m^2^, χ^2^ = 57.308, *P* < 0.001)

X-tail plots, constructed in, illustrated that the optimal cut-off point of BMI was 19 kg/m^2^ using minimum *P* value from log-rank ÷ 2 test, with the strongest discriminatory capacity (Fig. 1).

### Clinicopathologic characteristics of patients

A total of 302 T_2_DM patients with pT_1–4b_N_0–3b_M_0_ GC, underwent D_2_ radical resection, were collected for analysis with 132 deaths in a median follow-up of 87.6 months (range 63 to 114 months). A detailed description of the associations between BMI level and clinic-pathological characteristics were presented in Table 1. As anticipated, patients with low BMI (< 19 kg/m^2^) have high percentage of female, advanced T_4_ category (T_4a_ and T_4b_), advanced TNM category (stage III_A_, III_B_ and III_C_), and level of serum CEA (all *P* < 0.05). In the low BMI subgroup, the percentage of T_4_ category (79.3% vs 56.6%, P = 0.002) and stage_III_ (79.3% vs 56.1%, P = 0.002) was significantly higher in the low BMI subgroup than in the high BMI subgroup.

**Table 1:** Demographics and clinicopathologic characteristics of patients with gastric cancer

	BMI < 19 kg/m^2^ (n = 29)		BMI ≥ 19 kg/m^2^ (n = 273)		*P*-value
*H. pylori* infection					
Y	2	6.9%	17	7.2%	0.975
N	27	93.1%	220	92.8%	
Age	56.69 ± 13.1 57 (30–79)		58.81 ± 11.27 59 (23–82)		
Sex					
M	16	55.2%	180	75.9%	0.016
F	13	44.8%	57	24.1%	
CEA level	14.32 ± 4.53		11.58 ± 3.38		0.004
T category					
1a	–	–	22	9.3%	0.003
1b	–	–	22	9.3%	0.002^#^
2	3	10.3%	34	14.3%	
3	3	10.3%	25	10.5%	
4a	18	62.1%	127	53.6%	
4b	5	17.2%	7	3%	
N category					
0	4	13.8%	79	33.3%	0.301
1	7	24.1%	41	17.3%	0.082^$^
2	7	24.1%	41	17.3%	
3a	6	20.7%	45	19%	
3b	5	17.2%	31	13.1%	
TNM category					
IA	0		34	14.3%	0.213
IB	1	3.4%	23	9.7%	0.002^&^
IIA	3	10.3%	20	8.4%	
IIB	2	6.9%	27	11.4%	
IIIA	6	20.7%	31	13.1%	
IIIB	6	20.7%	37	15.6%	
IIIC	11	37.9%	65	27.4%	

M: male, F: female #: T4 VS T1-3, $: N3 VS N0-2, &: stage III VS stage I-II

### Survival analysis

**Fig. 2:**
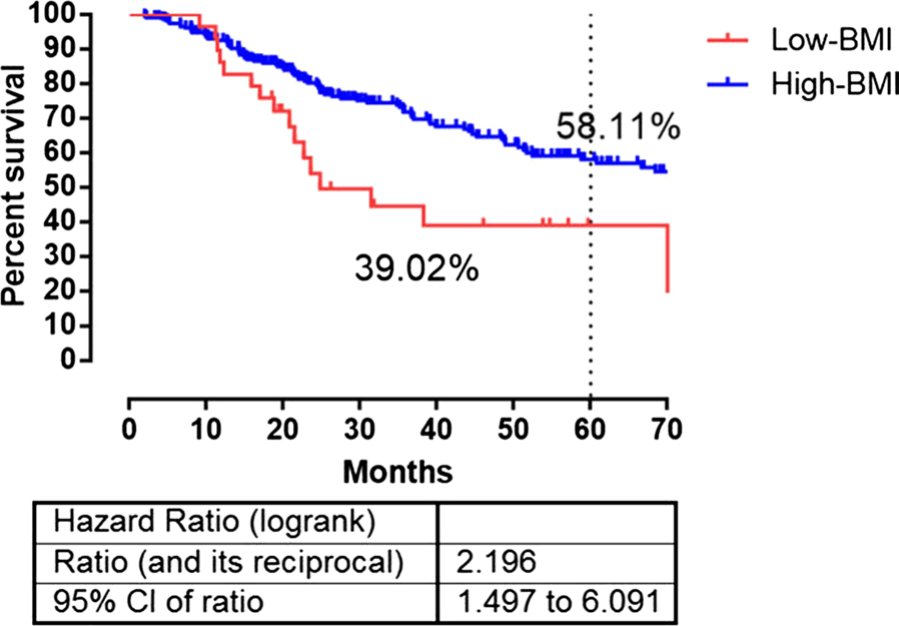
Survival analysis of gastric cancer patients with T_2_DM undergoing curative intent surgery. The P values for the survival comparison was determined by the log-rank test

**Fig. 3:**
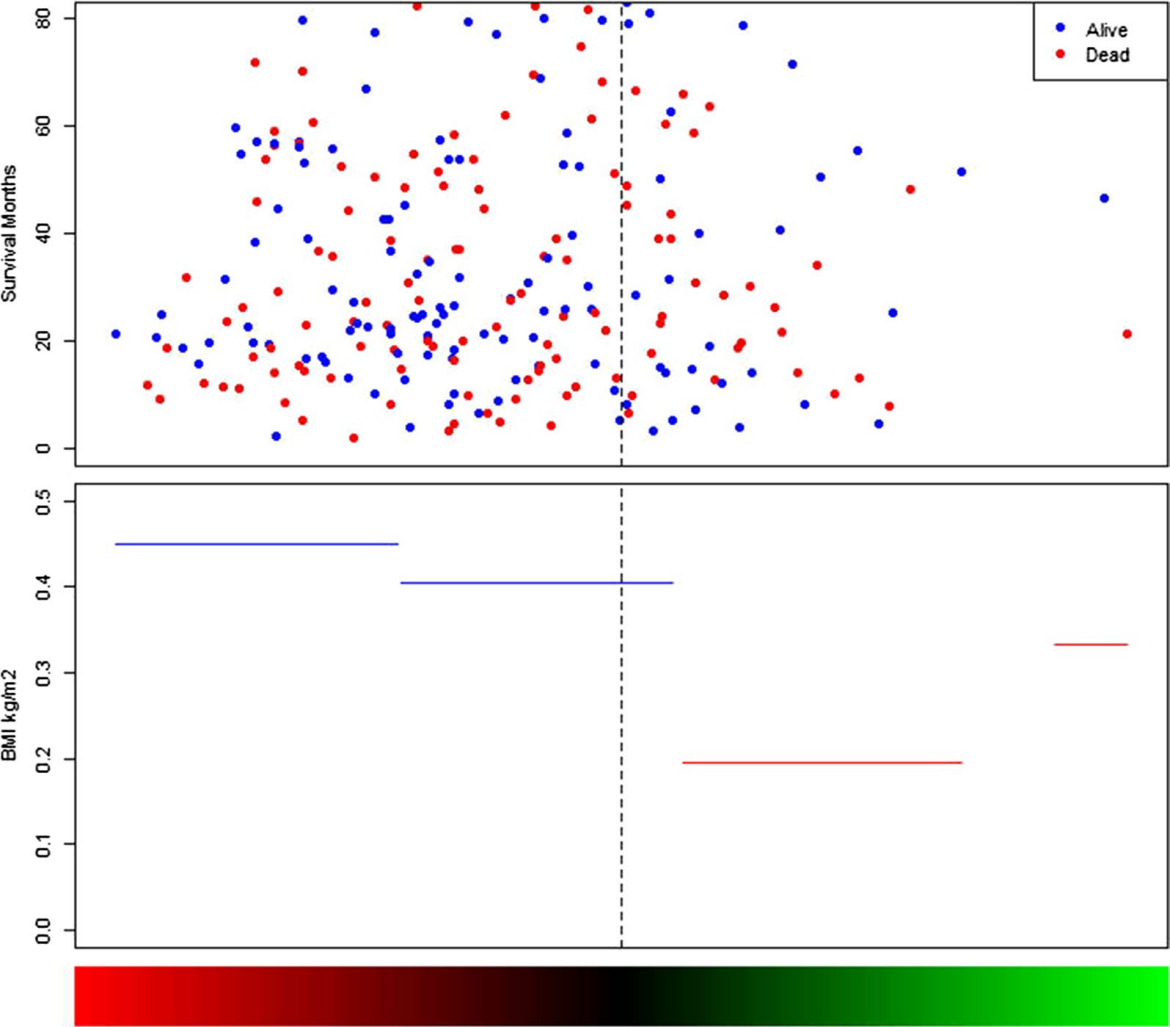
The risk score of BMI. The plot of HRs for BMI sharply increased as the number of BMI decreased

The 5-year OS of T_2_DM GC patients whose BMI less than 19 kg/m^2^ and more than 19 kg/m^2^ subgroups were 39.02% and 58.11%, respectively (Fig. 2). At the same time, the risk level of BMI was built using the linear combination of BMI with the estimated regression coefficients derived from the above Cox regression analysis as the weight to calculate the death risk score for each patient. Distribution of death and the survival status of stage I_A_–III_C_ GC were shown in Fig. 3. The plot of HRs for BMI sharply decreased as the level of BMI increased.

### Independent prognostic factors in the training cohort

**Fig. 4:**
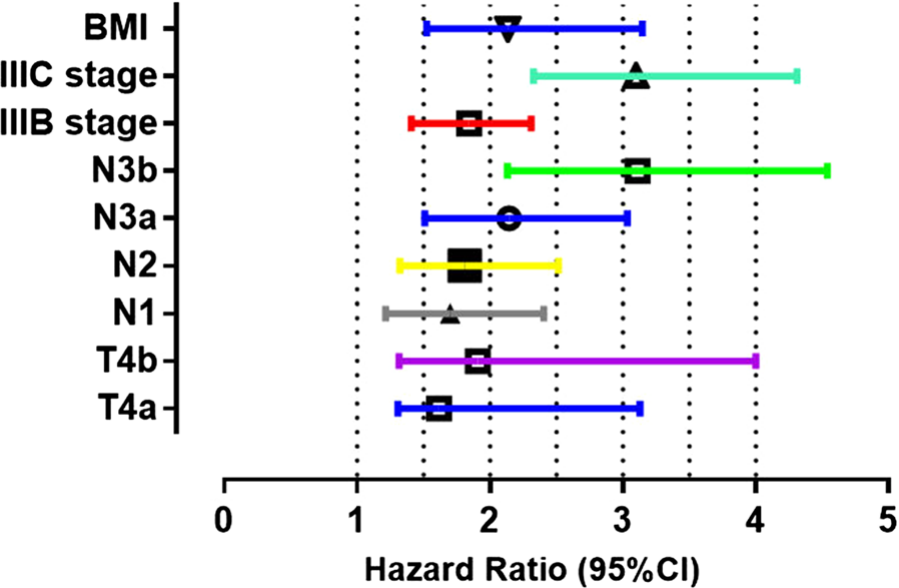
Cox multivariate analyses of prognostic factors for the overall survival of non-metastatic gastric cancer patients with T_2_DM after D_2_ lymphadenectomy

Variables considered significant in the multivariate analysis were entered in the Cox multivariate analysis. A total of six variables, including *H. pylori* infection (OR = 1.439), sex (OR = 0.943) have no statistical power (all *P* > 0.05). However, pretreatment BMI (OR = 2.136), III_B_ category (OR = 1.845), III_C_ category (OR = 3.101), T_4a_ category (OR = 1.617), T_4b_ category (OR = 1. 8), N_1c_ category (OR = 1.701), N_2_ category (OR = 1.812), N_3a_ category (OR = 2.145), and N_3b_ category (OR = 3.113), respectively (all P < 0.05) were proved independent in the multivariate Cox regression model (Table 2, Fig. 4).

**Table 2:** Cox proportional hazards multivariate regression analysis results

	B	SE	Wald	Sig	Exp(B)	95.0% CI used for Exp(B)	
						Low	Upper
*H. pylori* infection	0.364	0.368	0.977	0.323	1.439	0.699	2.959
Sex	− 0.059	0.254	0.053	0.818	0.943	0.573	1.552
BMI	0.036	0.005	43.388	0.000	2.136	1.525	3.147
TNM category							
IA					1 (reference)		
IB	− 7.243	36.242	0.040	0.842	1.153	0.514	2.584
IIA	− 7.871	36.272	0.047	0.828	1.211	0.704	2.130
IIB	− 9.443	36.313	0.068	0.795	1.331	0.654	2.411
IIIA	− 10.091	36.359	0.077	0.781	1.411	0.704	2.830
IIIB	− 10.396	36.399	0.082	0.000	1.845	1.410	2.312
IIIC	− 10.747	36.446	0.087	0.000	3.101	2.33	4.312
T category							
1a					1 (reference)		
1b	5.579	17.095	0.107	0.744	1.153	0.514	2.584
2	12.454	40.017	0.097	0.756	1.411	0.704	2.830
3	14.452	40.041	0.130	0.718	1.230	0.654	2.311
4a	14.850	40.063	0.137	0.012	1.617	1.308	3.130
4b	15.474	40.076	0.149	0.008	1.908	1.318	4.000
N category							
0					1 (reference)		
1	2.678	1.090	6.036	0.002	1.701	1.216	2.406
2	3.187	1.502	4.503	0.000	1.812	1.322	2.517
3a	3.918	1.919	4.167	0.000	2.145	1.510	3.034
3b	3.994	1.882	4.502	0.000	3.113	2.133	4.539

## Discussion

To the best of our knowledge, our work is the first one to systematically assess the clinical significance of BMI level in T_2_DM patients with non-metastatic GC. In spite of unclear underlying mechanisms, our study reveals that the pretreatment BMI is a significant indicator in predicting cancer-specific survival (CSS) in GC patients with T_2_DM. In the Cox multivariate analysis, pretreatment BMI under 19 kg/m^2^ was found to be an independent prognostic factor (OR = 2.136).

The important aspect in the intricate relation between the effect of BMI and GC is still unknown. Many investigation have been made on this relation, which harbor diverse results in terms of survival, pathological findings and surgical procedures [21, 27, 28]. Recent studies have reported a significantly decreased overall survival (OS) in underweight patients, defined as BMI under 18.5 kg/m^2^, who previously underwent gastrectomy due to GC [29], indicating a close correlation between low BMI and poor prognosis in GC patients. Consistently, our study found that the pretreatment BMI is a significant predictor of CSS in GC patients with T_2_DM. It was further confirmed that a preoperative BMI < 19 kg/m^2^ was a predictor of poor prognosis.

Lymph node involvement has been verified as the most independently essential factor for survival of GC [30–34], whose accurate evaluation largely depends on the sufficiency of lymphadenectomy [35]. In our data, N_3b_ category (OR = 3.113) was the most vital indicator, followed by N_3a_ category (OR = 2.145), N_2_ category (OR = 1.812), and N_1_ category (OR = 1.701). Curative surgery of GC is rather tough in case of T_4_ category, which includes tumor extension into serosa (T_4a_) as well as surrounding organs and tissues (T_4b_), which bears an unsatisfactory prognosis [36–38]. The 5-year survival rate of patients with T_4a_ GC has been reported to be rather low, 20% of whom pass away due to recurrence despite radical surgery of primary lesions. In the cohort, the percentage of T_4_ was up to 79.3% in patients with BMI < 19 kg/m^2^, and the corresponding data was 56.6% in the subgroup of BMI ≥ 19 kg/m^2^ (P = 0.002). In the cox multivariate analysis, T_4a_ category (OR = 1.617), T_4b_ category (OR = 1.908) were found to be independent risk factors. Pathologic TNM category is a helpful tool to predict prognosis in GC patients, nonetheless, a combination of preoperative BMI level can enhance predictive accuracy [39, 40]. In line with studies in other types of cancers, our findings demonstrated that low preoperative BMI (< 19 kg/m^2^) was a hazard factor for poor survival in patients with GC.

The phenomenon that obesity increase the risks of obesity-associated complications but decrease the mortality of patients which have critically illness is called “obesity paradox”. The underlying mechanism of obesity paradox still unclear but several explanations have been proposed. Some researchers doubt the protective effect of obesity in patients because they think that selection bias of patients exists. Subjective viewpoint of observational study and meta-analysis and the validity of BMI in patient’s evaluation may also confound the results of study about obesity paradox [41]. On the other hand, some mechanism about how adipose tissue exert protective effect have been proposed. They claimed that adipose tissues may be a marker of better health status of patients and be a source of energy and lipid-soluble nutrients. Marques et al. reported that adipose tissues may modulate the immune system of patients which may help with the improved survival [42]. The result of our study is another evidence that higher BMI may help patients get better prognosis.

Our work was a retrospective single-institute study, which was the major limitation. Anyhow, our work demonstrated for the first time that pretreatment BMI was associated with the prognosis of GC patients with T_2_DM. Specifically, a low pretreatment BMI predicted poor survival outcomes in GC patients with T_2_DM. The application of BMI is efficient, cost-effective and easy-calculated compared to other invasive procedures.

Collectively, our data showed that low preoperative BMI (< 19 kg/m^2^) was a prognostic factor for poor survival in patients with GC, and was useful in clinical practice and research design.

## Conclusion

Low preoperative BMI (< 19 kg/m^2^) was a poor prognostic marker for T2DM patients with pT_1–4b_N_0–3b_M_0_ GC.

## Data Availability

The datasets used and analyzed during the current study are available from the corresponding author on reasonable request.

## Abbreviations

BMI: Body mass index
T2DM: Type 2 diabetes mellitus
GC: Gastric cancer
WHO: World Health Organization
TNM: Tumor–node–metastasis
NCCN: National Comprehensive Cancer Network
